# Real-time monitoring of the budding index in *Saccharomyces cerevisiae* batch cultivations with in situ microscopy

**DOI:** 10.1186/s12934-018-0922-y

**Published:** 2018-05-15

**Authors:** Anna-Maria Marbà-Ardébol, Jörn Emmerich, Michael Muthig, Peter Neubauer, Stefan Junne

**Affiliations:** 1 Department of Biotechnology, Technische Universität Berlin, Ackerstrasse 76, ACK 24, 13355 Berlin, Germany; 2 SOPAT GmbH, Boyenstr. 41, 10115 Berlin, Germany

**Keywords:** In situ microscopy, *Saccharomyces cerevisiae*, Image detection, Budding index, Monitoring, Cell size, Morphology, Growth activity

## Abstract

**Background:**

The morphology of yeast cells changes during budding, depending on the growth rate and cultivation conditions. A photo-optical microscope was adapted and used to observe such morphological changes of individual cells directly in the cell suspension. In order to obtain statistically representative samples of the population without the influence of sampling, in situ microscopy (ISM) was applied in the different phases of a *Saccharomyces cerevisiae* batch cultivation. The real-time measurement was performed by coupling a photo-optical probe to an automated image analysis based on a neural network approach.

**Results:**

Automatic cell recognition and classification of budding and non-budding cells was conducted successfully. Deviations between automated and manual counting were considerably low. A differentiation of growth activity across all process stages of a batch cultivation in complex media became feasible. An increased homogeneity among the population during the growth phase was well observable. At growth retardation, the portion of smaller cells increased due to a reduced bud formation. The maturation state of the cells was monitored by determining the budding index as a ratio between the number of cells, which were detected with buds and the total number of cells. A linear correlation between the budding index as monitored with ISM and the growth rate was found.

**Conclusion:**

It is shown that ISM is a meaningful analytical tool, as the budding index can provide valuable information about the growth activity of a yeast cell, e.g. in seed breeding or during any other cultivation process. The determination of the single-cell size and shape distributions provided information on the morphological heterogeneity among the populations. The ability to track changes in cell morphology directly *on line* enables new perspectives for monitoring and control, both in process development and on a production scale.
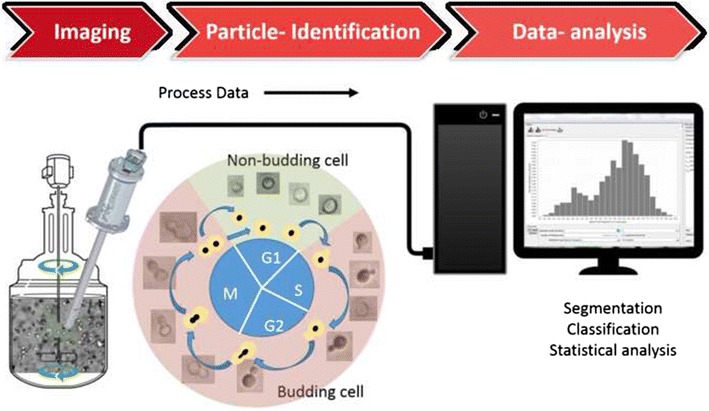

**Electronic supplementary material:**

The online version of this article (10.1186/s12934-018-0922-y) contains supplementary material, which is available to authorized users.

## Background

The morphology of single cells is traditionally measured with microscopy. Due to a certain relationship between form and function, the growth state of cells, and even the production performance can be investigated on the basis of cell size and other morphological features. As such features are determined *off line* or *at lin*e, they cannot be measured in real-time for the purpose of process monitoring.

Among the mostly applied methods to determine the growth activity is plating on solid media followed by incubation for several hours up to several days, or cell staining [1]. Microscopy is usually not connected to automated sampling, the achievement of a sufficient number of captured cells is time-consuming. Consequently, neither a representative sample is obtained, since only a few cells are counted at certain specific time points, nor the heterogeneity of the cell population is considered. If conventional microscopy is coupled to a sampling tube and flow cell, the sample is either affected by the conditions in the sample tube or the device has to be located very close to the reactor. This is often not applicable in daily laboratory practice.

Among automated methods for the characterization of a yeast population, flow cytometry (FCM) or cell counting is often applied. The morphological heterogeneity in a population can be measured as well with these methods. Moreover, FCM can provide further information beyond morphological features at the same time, e.g. total protein and DNA content measurements [2]. FCM was successfully applied to brewing yeast for the determination of the physiological state during propagation [3], and for the quantification of the vitality of cells before fermentation [4]. Partial least squares regression models were created using data from fluorescent propidium iodide staining microscopy and Coulter counter cell size distributions when cells were exposed to different stresses (temperature shift, acetate or furfural addition) [5]. Such methods are usually used for quality assessment, but have not become widely applied tools for process monitoring.

Other authors have used imaging microscopy [5, 6] or image cytometry of shake flask cultures [7, 8] for assessing the morphology of yeast cells. The acquisition of the images is conducted *off line*, but the particle recognition is usually automated. The cells are assumed to be elliptical, then the equivalent major and minor axes are determined. Further parameters such as cell size or volume are derived from this information. Image cytometry is a combination of image microscopy and the observation of light scattering data from cells that have been stained in order to directly assess viability. These methods are less time-consuming and avoid some of the typical errors of completely manual procedures, but certainly not all [9]. Sampling (automatic or manual) and staining is still required, when fluorescent markers cannot be applied, e.g. whenever targeted genetical modification is not possible.

If the morphology can be correlated with certain features of a culture, the single-cell size distribution can be used to investigate these without any further cell treatment. For example, morphological heterogeneity is affected by the age of cells and the status of the cell cycle [10]. Due to asymmetric division [11], budding of yeast cells can increase the morphological heterogeneity of a population. The usual variation of the composition and quality of complex media compounds [12] alters growth activity and population heterogeneity from batch to batch as well [13, 14]. Cultivation conditions influence this heterogeneity, since individual cells can react differently to them, e.g. at substrate limitation or during the accumulation of secondary metabolites. Single-cell monitoring, which generates statistically valid data, can therefore provide appropriate information on the status of a culture and contributes to an improved process and quality control.

The present study aims to achieve a further development stage by monitoring the maturation state of the budding yeast *Saccharomyces cerevisiae* with in situ microscopy (ISM) on a single-cell level. In the case of the budding yeast, the proportion of cells that are in the maturation state at a time (represented with the budding index, BI), can provide information about the growth vitality [15, 16]. An evolved version of a photo-optical probe, which was formerly applied in cultures of larger microbial cells like the heterotrophic microalgae *Cryptecodinium cohnii* [17], was used in yeast batch bioreactor cultivations for the first time. Automated image recognition was applied to differentiate between budding and non-budding cells on the basis of machine learning algorithms, and a correlation analysis was conducted in order to prove that data of ISM reflected well data of growth measurements throughout all process stages.

## Methods

### Yeast strain

The yeast strain *Saccharomyces cerevisiae* AH22 (MATa leu2-3 leu2-12 its4-519 can1) [18] was used for all experiments.

### Cultivation conditions

Cells were grown in buffered YPD medium at a pH-value of 5.5. The medium contained 2% of glucose, 1% of yeast extract, 2% of peptone, 1.4% of KH_2_PO_4_, 0.1% NH_4_Cl (all w/w) as described previously [18]. This complex medium was chosen rather than mineral salt medium in order to achieve conditions closer to industrial application.

Pre-cultures were grown aerobically in Ultra Yield™ Flasks (Thomson Instrument Company, VA, USA) at 25 °C and 250 rpm with 1% (v/v) of antifoam 204 (Sigma-Aldrich, Germany). Batch cultivations were conducted in a Biostat^®^ B plus stirred tank bioreactor (Sartorius AG, Germany). The temperature was set to 27 °C, the aeration rate to 1 vvm, and the stirrer speed to 400 rpm, respectively.

Cell growth was determined with the optical density at a wavelength of 600 nm (OD_600_) with a spectrophotometer (Ultraspec 3000, GE Healthcare, CT). Batch cultivations were inoculated so that the initial OD_600_ reached 0.3. The pre-culture was in the early log phase (OD_600_ = 4) at the time of inoculation. Baffled 250 mL shake flasks with non-invasive pH and DO sensors were used to record pre-culture conditions (PreSens-Precision Sensing, Germany). Alternatively, cell growth can be determined through the dry cell weight (DCW). 2 mL of culture were centrifuged for 10 min at 4 °C and 21,500×*g* in previously weighted 2 mL Eppendorf tubes, then washed with 2 mL of 0.9 g L^−1^ NaCl solution and centrifuged again under the same conditions as before. Then, the Eppendorf tubes were stored in a drying oven (75 °C) for 48 h and weighted.

The biological reproducibility of the three bioreactor cultivations was quantified with the standard deviation (σ) obtained between the values of the curve fit and of each experiment.

### *Off line* analysis

Every hour, a sample was taken for the measurement of cell growth and the quantification of extracellular metabolites. Cell growth was determined with the OD_600_ as described in the previous section. Samples for extracellular metabolite determination were filtered through a membrane filter with a pore size of 0.8 µm (Carl Roth, Germany). The supernatant was transferred to 1.5 mL Eppendorf tubes and immediately stored at − 80 °C.

Organic acids were quantified with an Agilent 1200 system, which was equipped with a refractive index detector and a HyperRez XP Carbohydrate H^+^ column (Fisher Scientific, Germany) as previously described [19].

### In situ microscopy

Cells were monitored in situ with the photo-optical probe SOPAT MM-Ho (SOPAT, Germany), which was installed directly in the bioreactor and dipped into the cell suspension. Another probe with stronger magnification, the SOPAT MM 2.1, was used through a bypass. The bypass was connected 2 h after inoculation.

**Fig. 1:**
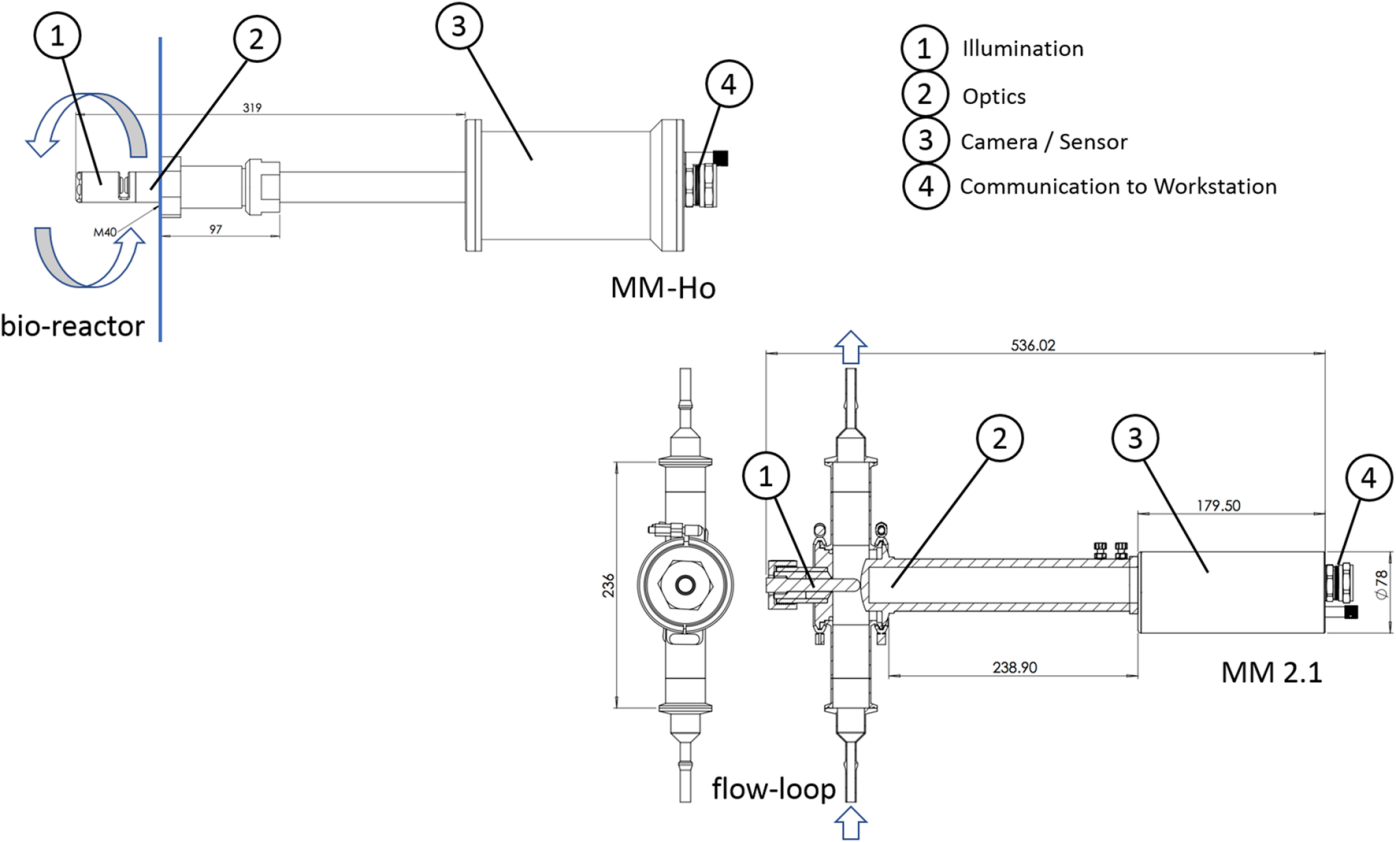
Sketch of the ISM devices: the probe MM-Ho was installed directly in the bioreactor, whereas the probe MM 2.1 was used in a bypass. The culture broth circulation is marked with arrows in each picture

Table 1 provides an overview of the main characteristics of both microscopic probes, Fig. 1 provides a schematic view of the devices. Both sensors used the same light source, but different optics and camera systems. The illumination is achieved by transmission, therefore the light source is located at the opposite side of the camera [20]. The light passes an adjustable distance (measuring gap) through the cell suspension. A short distance leads to the effect that light with a higher energy density re-enters the optical unit on the opposite side. More important is the decrease of obscuration due to overlapped cells within the measuring gap, especially at a high cell concentration. This results in images of higher contrast and an improved differentiation between objects and the background.

**Table 1:** Overview of the main characteristics of the MM-Ho and MM 2.1 probes

Parameter	MM-Ho	MM 2.1
Field depth [µm]	2.32	1
Camera	2750 × 2200 CCD with 19fps, 1”	2048 × 2048 CMOS with 26fps, 1”
Conversion factor [µm pix^−1^]	0.166	0.087
Interface	GigE Vision	GigE Vision
Magnification	26.6 ×	40 ×
Numeric aperture	0.1	0.55
Illumination	Transmission, Xenon flash lamp, 2.6 J, pulse duration 8 µs	Transmission, Xenon flash lamp, 2.6 J, pulse duration 8 µs
Measuring gap [µm]	40	50
Probe length [mm]	270	266
Probe diameter [mm]	24.5	50.0
Software version	SOPAT v1R.002.0053	SOPAT v1R.003.0092

Table 2 summarizes parameters of the image acquisition. As a result of the different optical configurations between the probes MM-Ho and MM 2.1, a number of settings were adjusted. Due to the different light transmission characteristics of the optics, the exposure time of the light needed to be increased by a factor of 10 for measurements with the MM 2.1 probe. The rate of captures were increased in parallel to a reduced field of view in order to obtain a sufficient amount of cells that were captured at each time point.

**Table 2:** Parameters of the image acquisition of the MM-Ho and MM 2.1 probe

Acquisition parameter	MM-Ho	MM 2.1
Image acquisition rate [min]	Each 3	Each 5
Exposure time [µs]	15	150
Stroboscope intensity [%]	5	5–12
Frames per trigger [-]	150	200

### Automated cell identification

**Fig. 2:**
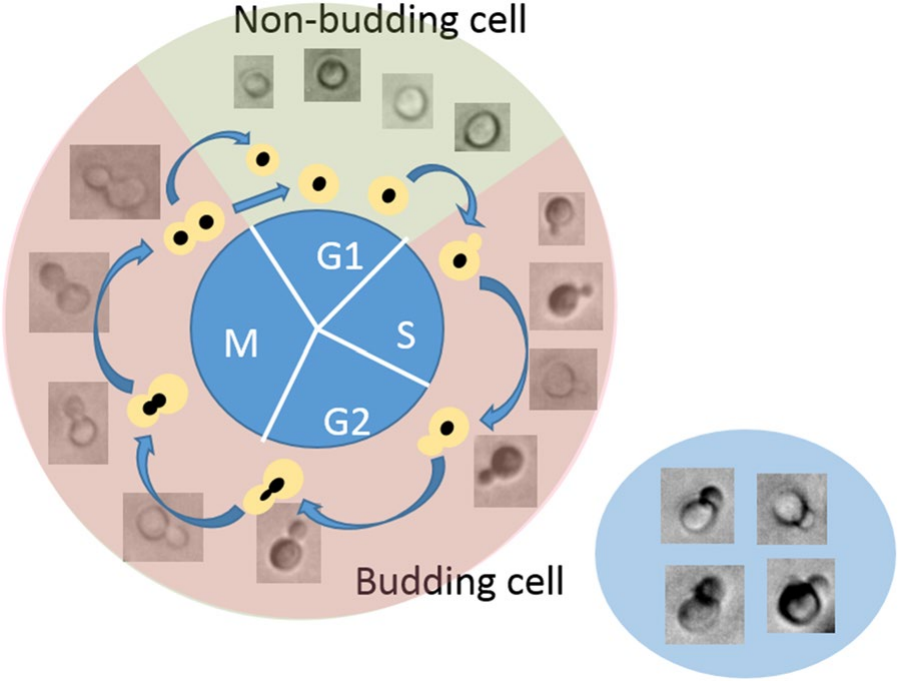
Classification of cells: non-budding (G1) states are depicted in front of a green background, budding cells (S, G2 and M) are depicted in front of a red background. Examples of overlapping cells that were excluded from the identification are shown in the blue ellipse

An artificial neural network (ANN) was trained for automated cell recognition. The first step was the annotation of the objects of interest, which were divided in two classes, budding and non-budding (including daughter) cells. As soon as a cell had a visible bud attached to the mother cell, it was considered as a budding cell. This large variability of automated recognizable particle sizes within the class of budding cells was feasible due to a flexible boundary detection. This was enabled through training the machine learning algorithm with annotated samples that covered the entire variability of the cell culture. Various examples of budding and non-budding cells are shown in Fig. 2. Agglomerates, and cells that were partly or completely out of focus were classified as background.

The annotated images served as training data for the ANN as previously described by Ronneberger et al. [21]. Afterwards, in order to exclude the falsely identified events, the detected and categorized objects, which were obtained from the ANN, were classified again. A normal Bayes classifier was trained with the labeled particles, from which a feature vector was created. This vector was a function of the area, convex area, eccentricity, equivalent diameter, perimeter and solidity.

Examples of the particle identification and classification are shown in the supplements (Additional file 1: Figure S1). The portion of false positive (particles erroneously detected as cells) and false negative (cells that were not recognized as such) was approx. 5% of the sum of correctly recognized and false negative counts as determined by manual annotation for the captures of the probe MM-Ho (Additional file 1: Table S1) and slightly higher for captures of the probe MM 2.1 (Additional file 1: Table S2). The budding index (BI) was automatically calculated based on the classification of budding and non-budding cells.

### Reliability of the automatic cell identification

**Fig. 3:**
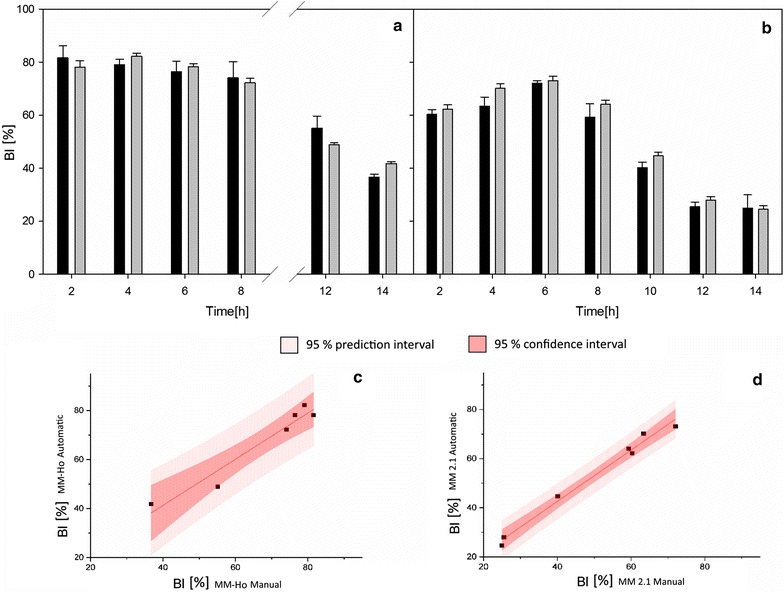
Comparison between the budding indices obtained with ISM (black bars) and with manual counting (gray bars) with the respective standard deviations for the probe MM-Ho (**a**) and the probe MM 2.1 (**b**). The standard deviation of the manual counting was obtained from two mean values from a sample size of between 100 and 200 cells. The standard deviation of the automated cell detection is calculated as difference between values of a fit (spline function) and values of a capture event. Linear correlation between the manual and automatic recognition for the probe MM-Ho (**c**) and the probe MM 2.1 (**d**)

In order to proof the reliability of the cell detection, a manual counting of budding and non-budding cells was performed with captures of the two probes. The automated cell detection has a lower standard deviation than the manual detection, both recognition methods yield similar results (Fig. 3a, b). The correlation between the BI derived from data of automated and manual cell detection was R = 0.98 for the MM-Ho probe (Fig. 3c) and R = 0.99 for the MM 2.1 probe (Fig. 3d). In case a sample was measured three times, a coefficient of variation of less than 0.15% was achieved. The divergence in the BI of captures from the probes MM-Ho and MM 2.1 is seen in both manual and automated cell detection. This divergence might be due to differences of the pre-culture (biological divergence), but also due to the differences in the bypass unit (technical divergence). Due to the setup of the bypass, it is considered to be unlikely that yeast cells are affected in such a short time of 60 s at the given concentration. The impact is lower than at *off line* microscopy anyway, which is the only reliable reference method. Therefore, any influence would be hardly detectable, if it is less than at the sample treatment for *off line* measurements. In any case, the same dynamics of metabolic concentrations and morphologic cell features were observed.

A certain portion of budding cells are identified as non-budding cells, if (1) either the bud is hidden by its mother cell (optical shadow), or (2) if the bud is situated directly in front of it. This percentage can be approximated under consideration of the portion of the surface area of the mother cell, in which a daughter cell (bud) is completely hidden (A_hd_). Finally, the relation between the total surface (S_m_) and the A_hd_ multiplied by two will provide the probability of false positive detections in non-budding cells (X_Fnb_). If X_Fnb_ is derived as explained in the supplementary materials (Additional file 1: Figure S2), a maximum of 4% of all cells will be classified as non-budding, although they should actually be classified as budding cells. An even distribution of cells towards the optical plane is assumed. However, it is likely that cells are oriented towards the direction of flow. In this case, the bud will likely be located in orthogonal direction towards the optical plane. Thus, the proportion of buds in the optical shadow or directly in front of the mother cell is lower than assumed under a normally distributed cell orientation. Since the error is systemic and similar across all cultivations and also occurs with the usual *off line* light microscopy, it is considered to be negligible for the further discussion.

### Sample size and sample concentration

In order to ensure that a representative sample of the cell population was measured, a sensitivity analysis from each cell class (budding and non-budding) was performed, as both classes had a different grade of heterogeneity (data not shown). A certain heterogeneity is obtained, because budding particles vary in size due to the ongoing budding process. A higher heterogeneity requires more data for training. It was taken care that the number of cells that are identified at each time point exceeded the required number to gain a reproducible value of the mean cell Feret diameter and the Dv90 (the cell size, which encounters 90% of the detected cell sizes).

### Morphological parameters

The morphological parameters that were obtained from the ISM were the cell Feret diameter (d_F_) and the aspect ratio (AR). A certain d_F_ of a particle is calculated as the difference between the maximum and the minimum length of the particle projection on a unit vector with a certain rotation. The minimum, maximum and mean d_F_ are estimated by rotating the unit vector from 0° to 180° by 16 steps. Then, the smallest, largest and mean diameters are determined according to ISO norms. The aspect ratio is obtained by dividing the minimum through the maximum d_F_.

In order to reduce the influence of outliers, the median of the minimum, maximum or mean d_F_ of all cells is shown in the manuscript. Moreover, the interquartile range (IQR), which is the difference between the 75th percentile, also denoted as third quartile (Q3), and the 25th percentile, also denoted as first quartile (Q1), is provided to indicate the variability around the median.

## Results

### In situ monitoring of the budding index

**Fig. 4:**
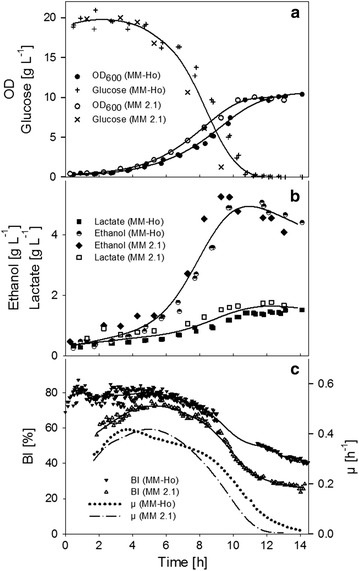
Performance of *S. cerevisiae* batch cultivations: **a** OD_600_ of cultures monitored with the probe MM-Ho and MM 2.1 and the respective glucose concentration, **b** ethanol and lactate concentrations. The standard deviation between experimental points and curve fits (spline function) are 0.46 (OD_600_), 1.2 g L^−1^ (glucose), 0.3 g L^−1^ (ethanol), and 0.2 g L^−1^ (lactic acid concentrations). **c** Budding index as determined with the probe MM-Ho and MM 2.1, and the specific growth rate. Experimental data is represented with dots, curve fits with straight lines

Three glucose-limited aerobic batch cultivations of *S. cerevisiae* were conducted. In addition to the standard *off line* sampling for the investigation of cell proliferation and the metabolite concentration profile (Fig. 4a, b), ISM was used to obtain information on the growth vitality of yeast cells. Two cultivations were monitored with the probe MM-Ho, and one with the probe MM 2.1.

**Fig. 5:**
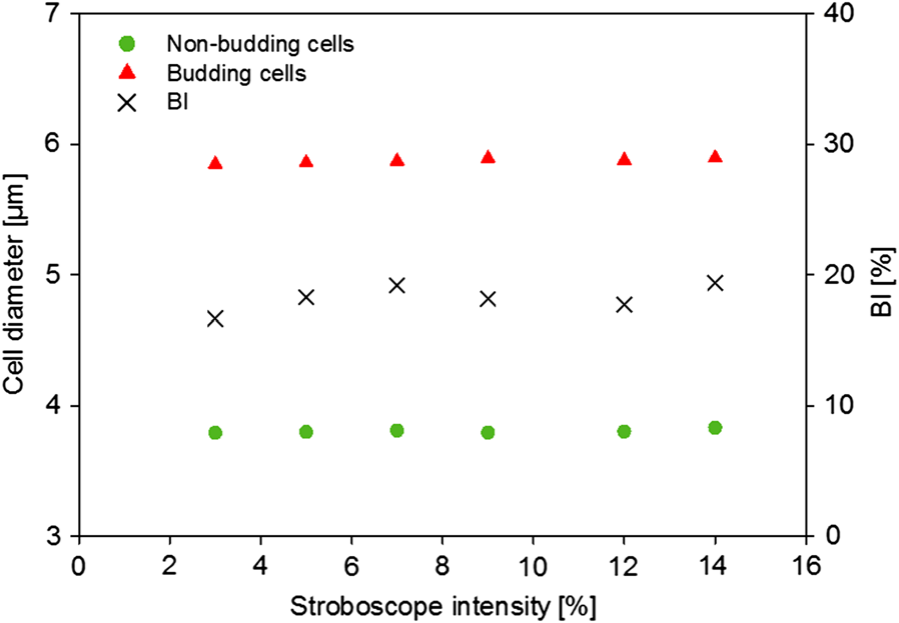
Yeast cells of the stationary phase measured with six different stroboscope intensities. The median of the mean d_F_ of budding and non-budding cells, and the budding index is depicted

The increment of the cell concentration over time of either OD_600_ = 6.2 or 2.8 g L^−1^ of DCW exceeded the threshold value for a suitable cell identification due to numerous overlapping objects. For an in situ application, however, the probe must cover a common concentration range. In order to achieve this objective, two parameters were tested for their robustness: (1) the modification of the focus plane, and (2) the stroboscope intensity. The first one influenced the sharpness of the separation of a cell from the background, which has an influence on the determination of the cell size. It must therefore remain constant during an application. However, it has been demonstrated that adjusting the stroboscope intensity did not affect the results. Although captures were gained at different stroboscope intensities, neither the BI nor the cell size of budding and non-budding cells were affected (Fig. 5). A further development to automate the adjustment of the stroboscope intensity to the increase of the cell concentration is in progress.

Monitoring with the MM 2.1 probe (with a higher magnification) was performed with an adjusted stroboscope intensity. In parallel to the increasing cell concentration, the intensity was increased from 5 to 12% after 9 h. This allowed for a proper cell detection throughout the entire process.

All cultures performed similarly in terms of growth, production and consumption rates as well as cell morphology (Fig. 4). The two cultivations, in which monitoring was conducted directly *in line* in the cell suspension, were almost uniform, and a third cultivation, in which ISM was applied in a bypass, showed only minor deviations from the previous experiments (values of the standard deviation are listed in the legend of Fig. 4).

The patterns were typical for aerobic cultivations with yeast. By using a pre-culture that was inoculated during its early exponential phase, a growth rate of 0.3 h^−1^ after 1 h of bioreactor cultivation was achieved, followed by an exponential growth phase, in which a maximum rate of 0.42 h^−1^ was reached (Fig. 4c).

In each of the growth phases, a different BI was obtained. The BI increased to a maximum of 80% in the first hour after inoculation. Afterwards, a reduction in the BI was observed, which can be attributed to mitosis, as the proportion of daughter cells from freshly saturated cells increases.

As soon as the culture entered the exponential phase, the BI began to decline due to an accelerated proportion of mature daughter cells. It decreased linearly at a rate of − 4.4 ± 0.13% h^−1^ up to about 9 h after inoculation. The trend in the BI decreased to 22% when the glucose concentration was close to limitation (measured with the probe MM 2.1). After the shift to ethanol consumption, the proportion of budding cells decreased, while maturation slowed down and only a few cells entered the S-phase. While the development of the BI is uniform among the different probes, which were applied, the absolute value differs. This is most likely due to an improved recognition of cells, which belong to the class of small non-budding cells due to improved optics of probe MM 2.1. This can be seen in Fig. 7.

The BI is well correlated with the growth activity of cells, as demonstrated by a cross-calibration correlation analysis (provided in the supplementary material). This correlation applies to more or less all growth stages with a coefficient of determination of R = 0.99 (Additional file 1 Figure S3).

**Fig. 6:**
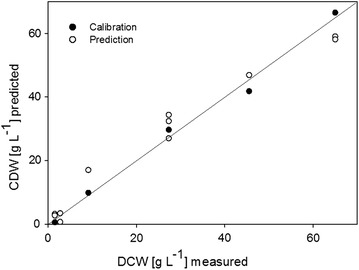
Linear correlation between the DCW as measured *off line,* and the DCW as predicted with ISM. Depicted are the values used for calibration and prediction

A maximum DCW of 5 g L^−1^ was achieved during batch experiments. No more than 12% of the light capacity of the stroboscope was used. Therefore, there is a potential to monitor higher concentrated cell suspensions. A concentration range of 3 to 65 g L^−1^ was tested by adjusting the stroboscope light intensity. Then, the images were processed as follows: first, a Laplace filter was applied to the original image. Then, the output was normalized with the average image brightness at the adjusted stroboscope intensities. Afterwards, the predicted concentration was calculated with the most suitable feature combination for the best fit of true concentrations. The correlation between the measured DCW and the predicted DCW was R = 0.97 (Fig. 6). Nevertheless, in order to achieve reliable results of the BI at higher cell densities, a further annotation and training of the ANN is suggested.

### Growth dynamics and population heterogeneity

**Fig. 7:**
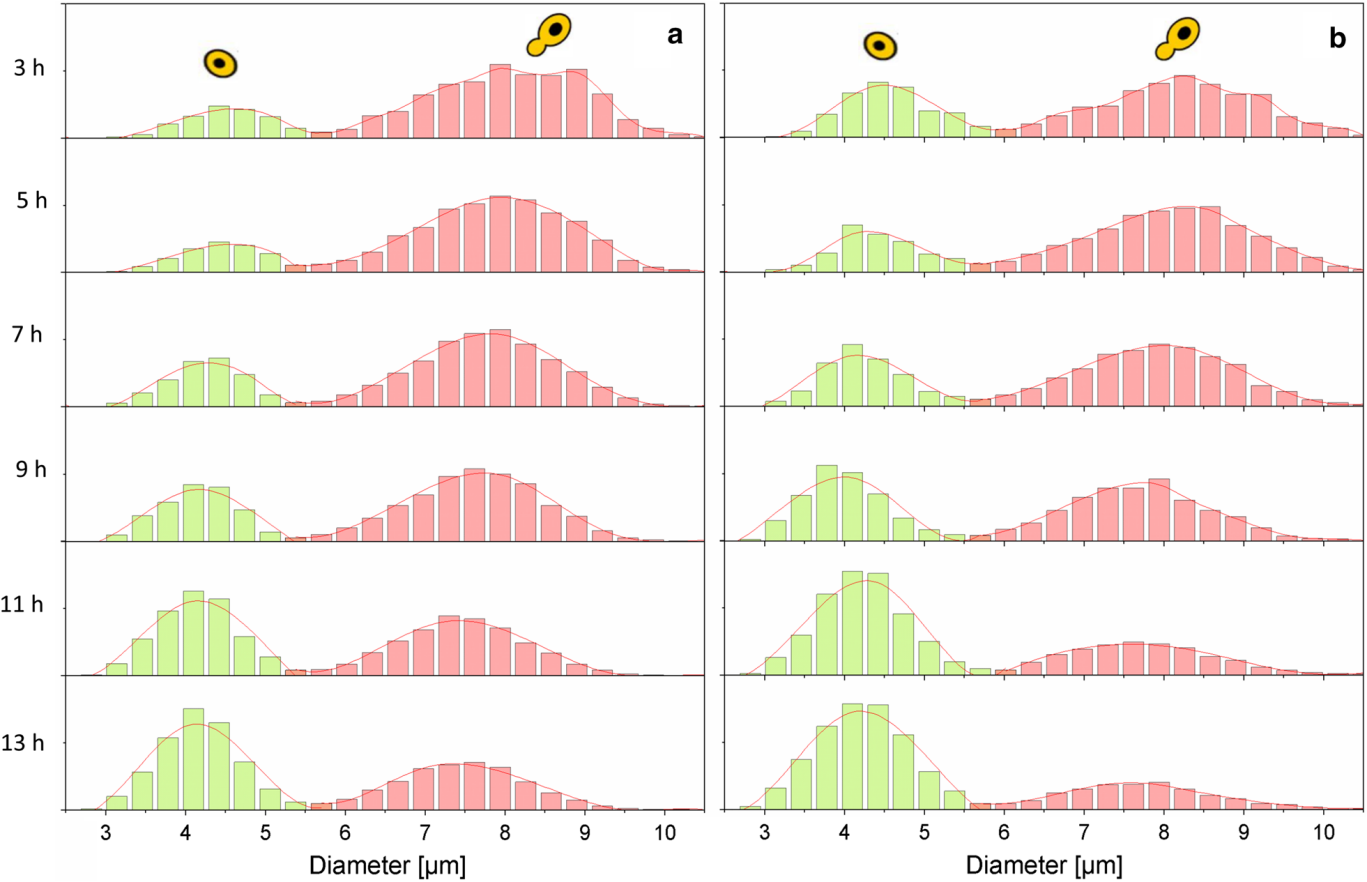
Single-cell size frequency distribution of the max. d_F_ for cells classified as budding (red) and non-budding (green) measured with the probe MM-Ho (**a**) or MM 2.1 (**b**)

The heterogeneity of the population can be studied using the morphological parameters determined by the ISM. The sample was divided into two populations, budding and non-budding cells. This resulted in a bimodal distribution. Figure 7 shows the unicellular size distribution in relation to the max. d_F_ of some selected time points during the batch cultivations. The distribution was wider in the early stage of the exponential phase (3 h). The portion of small cells increased after 9 h of cultivation, when cells reached the post-diauxic phase, while the distribution between small and large cells remained rather constant between 3 and 7 h of cultivation. There existed a similar size distribution over a certain time period, when the change of substrate availability, byproduct formation and other changing factors do not have an impact on growth and vitality.

**Fig. 8:**
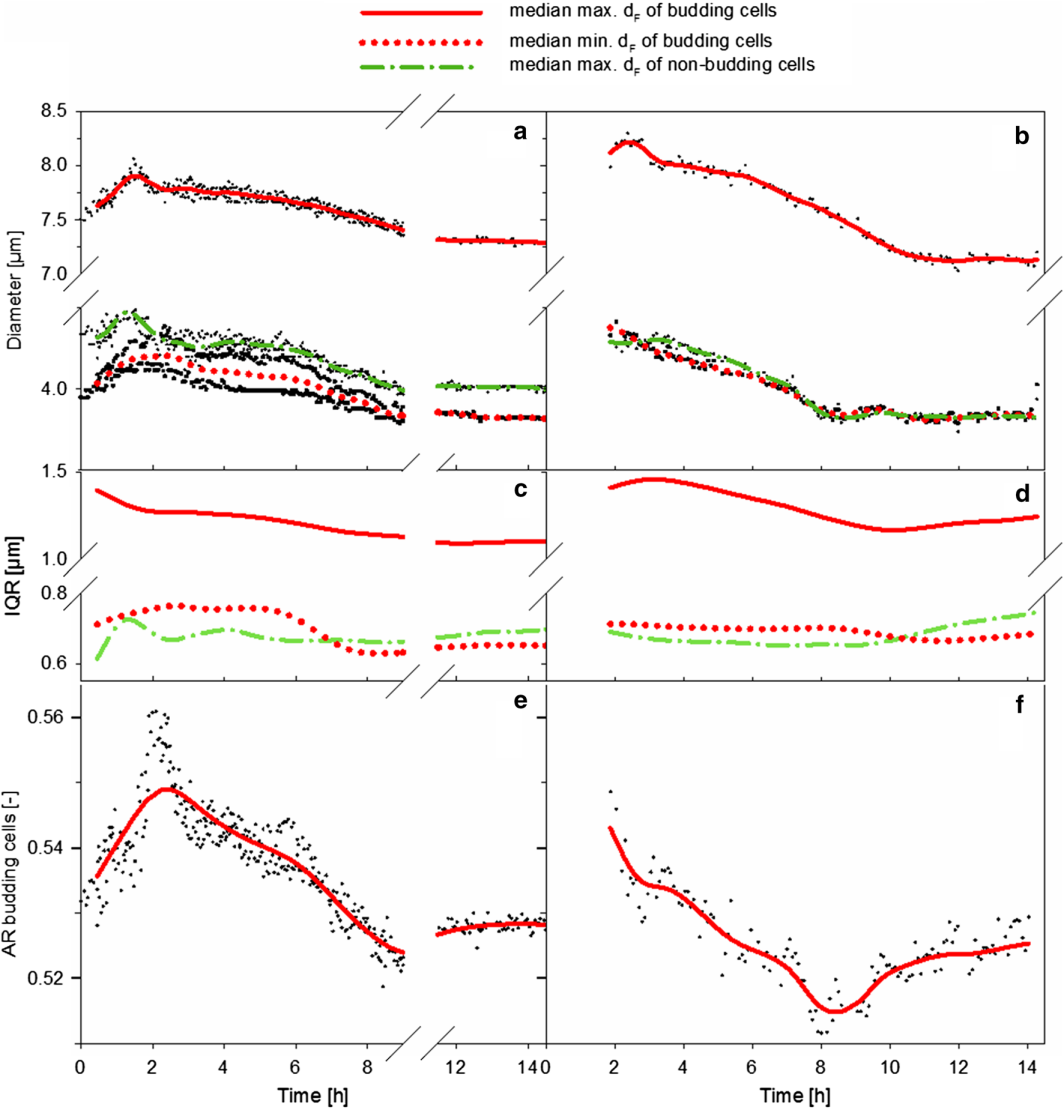
Variation of morphological parameters. Values in the left column were obtained with the MM-Ho probe, values at the right column with the MM 2.1 probe. **a**, **b** Evolution of the median of the max. d_F_ and the min. d_F_. **c**, **d** Variation of the interquartile range (IQR) of the median of the max. d_F_ and the min. d_F_. **e**, **f** Evolution of the aspect ratio (AR) for budding cells, that is the ratio between the median of the min. d_F_ and the median of the max. d_F_

The size development during a cultivation is shown in Fig. 8. Since the daughter cell is always smaller than its mother cell, the difference of the minimum and maximum d_F_ of budding cells provides information about the bud size. The mother cell usually remains almost invariably large during the budding phase, the d_F_ of mother cells is rather a value between the minimum d_F_ of budding cells and the maximum d_F_ of the non-budding cell fraction. The maximum d_F_ of the non-budding cells remains almost constant during the growth phase, while the minimum cell size of budding cells decreases in parallel to a decreasing growth rate. The cell size is affected by many parameters, among them are internal metabolite and ion concentrations, lipid, protein and RNA contents. These are steadily changing while growth decelerates. The appearance of smaller budding cells might thus be an early indicator for growth retardation. The heterogeneity in the lag phase is greater than in the post-diauxic phase, as a broader cell-to-cell variation is probably due to the stress response after transfer and inoculation than during glucose starvation, in which cells likely respond in a similar manor. The homogeneity of budding cells increased during cultivation (Additional file 1: Figure S4).

The AR of the non-budding cells remained almost constant (~ 0.9), since these cells are preserved as almost perfect spheres. From the moment on when the cells entered the exponential phase, the AR of budding cells probably decreased due to an increase in cell size during the maturation phase under elevated growth [22]. When diauxic growth occurred, the AR increased due to a reduced bud size due to the retardation of bud growth. It seems that the cell cycle stagnated at the same time, while the BI changed only slightly. All other morphological parameters remained constant, so that hardly any cell entered the S-phase. Multi-budding yeast cells were hardly observable among all cultivations and therefore neglected during cell recognition.

## Discussion

Many efforts have been made to develop in situ microscopes for the application in bioprocessing [23, 24]. Initially, probes (Type III XTF, Sartorius and Hannover Univ.) required mechanical sampling, or a bypass measurement [25]. Previous studies used ISM without mechanical sampling techniques for the determination of the biomass concentration and the cells’ volume. The cell concentration was examined with a further developed version of ISM type XTF. The biomass concentration of the yeast *Pichia pastoris* was monitored up to a concentration of almost 80 g L^−1^ with a standard deviation of less than 12% [26] (similar to results shown in this paper, 65 ± 4 g L^−1^). The cell size variation, which was influenced by osmotic stress responses, was assessed [27]. Cell identification was conducted with template matching and the resembling of circles. Aggregates were ignored like in our study. The volume of cells (30–38 µm^3^ during the batch phase) indicate that the detection was rather restricted to non-budding cells.

In order to distinguish between budding and non-budding cells as performed in this study, it was assumed that the cell projection is an ellipse and the relationship between the major axis and minor axis can be used to classify the maturation state (that is the aspect ratio, as shown in the Results section, or the elongation). Thus, it yields an approximation of the BI [6]. Therefore, the AR or elongation value need to be set to discriminate both maturation states. Consequently, budding cells may be considered as non-budding cells at the beginning of the S-phase. However, the value selected by Coelho, et al. (elongation = 1.5) correlates well with the data presented in this study.

ISM was not affected negatively by agitation as long as the power input was sufficient to generate a certain flow through the measurement gap. Then the image acquisition frame rate was adjusted in order to guarantee that cells from a previous frame will not appear in the following frame. Captures including bubbles were ignored for further image analysis. Undissolved particles of complex media are not influencing cell detection since the ANN approach will recognize those particles as background. This was applied in this study as complex media with yeast extract and peptone was used. However, threshold concentrations will exist, which does not allow for a precise measurement anymore, but this depends on many factors and has to be evaluated in each specific case. One benefit of any automated cell detection method is the consideration of a large number of cells within a short time. The minimum sample size, which is required to obtain a representative cell size distribution (n) of a population (N), can be determined with Eq. 1. This approximation is based on the assumption that N is much larger than n, and that n is normally distributed [28]. The desired accuracy (e) was set to 5% of the variance of the max. d_F_ of budding and non-budding yeast. The admitted error was assumed to be α = 5% among the number of annotated cells n. A Gaussian distribution of the cell size of each class (z_1−α/2_ = 1.96) was considered. The amount of cells that needed to be identified from each class at each sample point under the assumptions described above is shown in Table 3.

1 $$n = \left( {\frac{{\sigma z_{1 - \alpha /2} }}{e}} \right)^{2}$$

**Table 3:** Sample size to obtain representative data of a population with the probes MM-Ho and MM 2.1

	MM-Ho		MM 2.1	
	Non-budding	Budding	Non-budding	Budding
Variability (σ) [µm]	0.55	0.89	0.52	0.95
Accuracy (1−α) [µm]	0.05	0.06	0.04	0.07
Sample size (n) [-]	502	768	633	794

The recognized amount of cells exceeded these cell numbers at all analyses.

The processing of captures lasted about 16 s with the probe MM-Ho. Hence, the estimated total process time for a sample point with 150 images was approx. 40 min. The higher magnification reduced the time of image post-processing with the probe MM 2.1. Only 2.5 s were required to process an image, in total approx. 8 min for a sample point of 200 captures. Consequently, the method is assumed to be suitable for real-time monitoring and control of a bioprocess.

A study by Rupes et al. observed a systematic error at automatic image recognition validation [29], as the deviation between manual and automatic detection increased when large quantities of buds were analyzed. No systematic error was observed in the present study. Although the divergence between manual and automatic detection at some points in time is up to 14%, it remained under 8% on average (Additional file 1: Tables S1 and S2).

Population heterogeneity can make a difference in the performance of a culture. Therefore, monitoring of the heterogeneity is crucial as it can influence the robustness and productivity of a bioprocess [30]. A certain cell size have to be reached for the initiation of budding and DNA replication [15]. The same critical cell size applies to all daughter cells, while it increases with age in parental cells. The heterogeneity among budding cells was reduced during the growth phase, while it was higher in the lag phase. As recent studies have shown, bet-hedging mechanisms may be the reason for prolonged growth delay periods due to the formation of subpopulations with different phenotypes when cells cope with environmental stress, as it occurs after inoculation [31].

The determination of yeast quality was often determined by the viability and vitality of cells. However, viability is not clearly defined and can be seen as a continuum of cell activity, from very active to very inactive cells, which is unacceptable for cultivation [4]. Real-time monitoring of growth activity on a single-cell basis is achieved with ISM, so that it does not rely on an average value, but provides the possibility for a continuous observability of the population heterogeneity based on growth activity. Until now only animal cell structures, as detected with ISM, were related to cell viability [32]. The proper detection of smaller cells like many bacteria remains still a challenge and clearly limits currently ISM for the application in such bioprocesses. Although some studies have investigated the cell concentration of bacteria like *Escherichia coli* [33], the determination of the dried biomass concentration was only recently performed on the basis of a grayscale intensity measurement of captures. Single-cell analysis as described for cell line cultivation, algae or yeast has not been conducted yet for bacteria. Further experiments are required to investigate the limits of application at high cell densities at various complex media compositions.

## Conclusions

ISM was applied successfully to monitor growth and budding activity in yeast batch cultures. In addition to growth information, the heterogeneity among the population of budding yeast cells can be quantified as well. The measurement can be performed directly in the reactor during the cultivation period by means of a photo-optical sensor in conjunction with an automated image analysis. Although other techniques can also provide data about cell size distributions, imaging microscopy gain data about the shape and any potential structural segregation of individual cells. In addition, the use of accelerated image recognition for process control is conceivable. In order to further improve applicability to differentiate cell structures, e.g. at intracellular product accumulation, the application of a higher resolution and the consideration of overlapping cells are currently under development.

## Additional file

**Additional file 1.** Tables and Figures.

## Abbreviations

ANN: artificial neural network
AR: aspect ratio
BI: budding index
d_F_: cell Feret diameter
DO: dissolved oxygen
DCW: dry cell weight
FCM: flow cytometry
ISM: in situ microscopy
IQR: interquartile range
