# Supplementary material for: Real-time monitoring of the budding index in Saccharomyces cerevisiae batch cultivations with in situ microscopy
Source: Microb Cell Fact. 2018 May 15;17:73. doi: 10.1186/s12934-018-0922-y (PMC5952372; doi:10.1186/s12934-018-0922-y)
Supplement: Supplementary file 1 — Additional file 1. Tables and Figures. [file 12934_2018_922_MOESM1_ESM.docx]

Additional file 1

Table S1. False positively (PF) and false negatively (FN) classified cells, if automated and manual identification are compared (MM 1 probe)

| **Time [h]** | **Class** | **Identified cells** | **FP counts** | | **FN counts** | **FP [%]** | **FN [%]** |
| --- | --- | --- | --- | --- | --- | --- | --- |
| 2 | B | 146 | 1 | 5 | | 0.7 | 3.3 |
|  | NB | 41 | 3 | 0 | | 7.9 | 0.0 |
| 4 | B | 181 | 4 | 4 | | 2.2 | 2.2 |
|  | NB | 41 | 2 | 4 | | 4.7 | 9.3 |
| 6 | B | 313 | 18 | 10 | | 5.9 | 3.3 |
|  | NB | 82 | 2 | 2 | | 2.4 | 2.4 |
| 8 | B | 473 | 19 | 30 | | 3.9 | 6.2 |
|  | NB | 183 | 10 | 6 | | 5.6 | 3.4 |
| 12 | B | 281 | 13 | 28 | | 4.4 | 9.5 |
|  | NB | 320 | 13 | 6 | | 4.2 | 1.9 |
| 14 | B | 288 | 14 | 18 | | 4.8 | 6.2 |
|  | NB | 364 | 9 | 11 | | 2.5 | 3.0 |

Table S2. False positively (PF) and false negatively (FN) classified cells, if automated and manual identification are compared (MM 2.1 probe)

| **Time [h]** | **Class** | **Identified cells** | **FP counts** | | **FN counts** | **FP [%]** | **FN [%]** |
| --- | --- | --- | --- | --- | --- | --- | --- |
| 2 | B | 77 | 3 | 6 | | 4 | 8 |
|  | NB | 44 | 1 | 5 | | 2 | 10 |
| 4 | B | 153 | 5 | 18 | | 3 | 11 |
|  | NB | 63 | 0 | 10 | | 0 | 14 |
| 6 | B | 184 | 7 | 6 | | 4 | 3 |
|  | NB | 55 | 0 | 4 | | 0 | 7 |
| 8 | B | 87 | 6 | 9 | | 7 | 10 |
|  | NB | 61 | 0 | 9 | | 0 | 13 |
| 10 | B | 100 | 10 | 12 | | 10 | 12 |
|  | NB | 149 | 1 | 10 | | 1 | 6 |
| 12 | B | 47 | 2 | 3 | | 4 | 6 |
|  | NB | 116 | 1 | 7 | | 1 | 6 |
| 14 | B | 50 | 6 | 4 | | 13 | 8 |
|  | NB | 145 | 5 | 7 | | 3 | 5 |

Figure S1


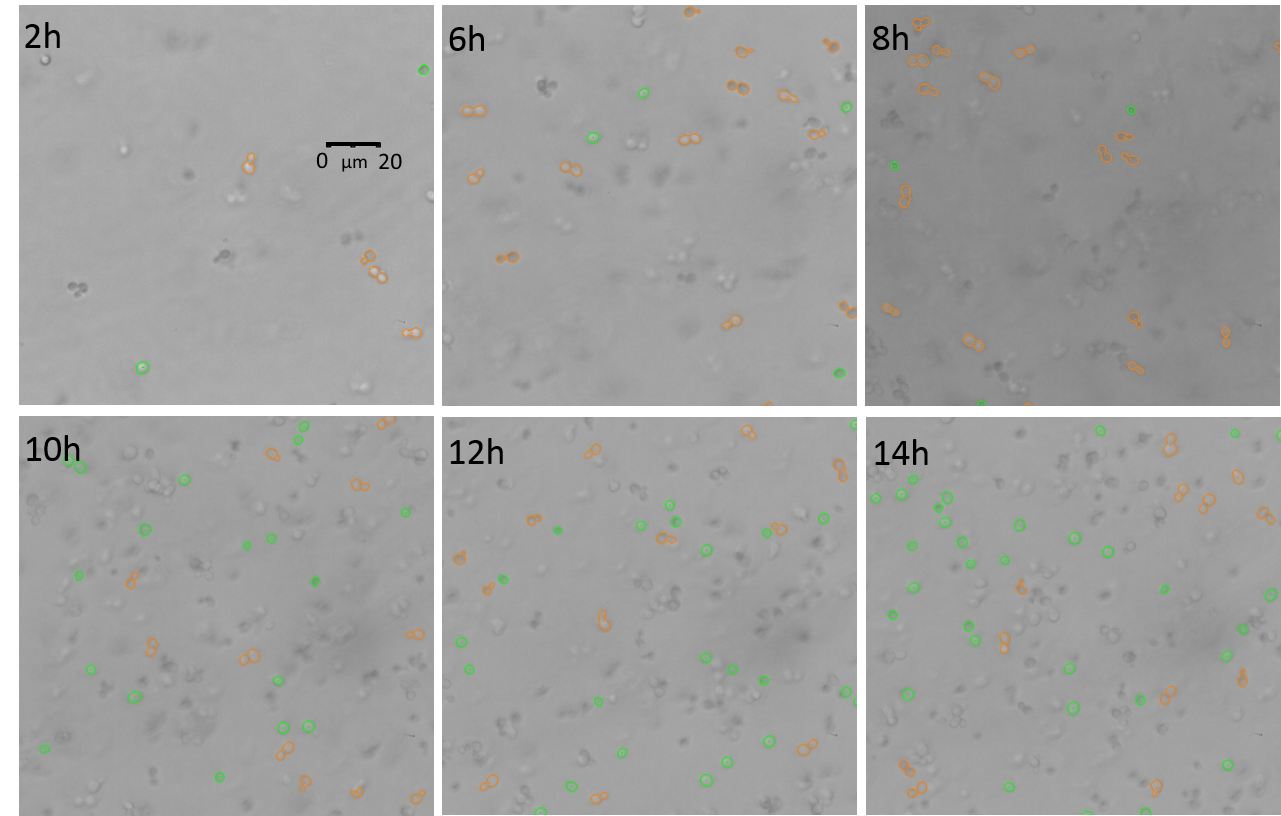


Images as acquired with the probe MM 2.1 during the cultivation. Budding cells are shown in red and non-budding cells in green as classified with the automated cell detection.

*Determination of the portion of budding cells, which are detected as non-budding cells*

Several of the parameters, which are used in the following, are depicted in figure S1. In order to estimate the portion of wrongly classified cells, a perfect circularity of the mother cell and the bud is assumed. Firstly, the surface of the mother cell (S_m_) is calculated (Eq. 1). The median value of the max. d_F_ of the non-budding (d_Fmax,nb_) is considered as diameter of the sphere with the respective radius (r_m_):

$S_{m}=\pi d_{Fmax,nb}^{2}$ (1)

Then, the diameter of the daughter cell (d_d_) is estimated (Eq. 2):

$d_{d}=d_{\mathrm{Fmax},b}-d_{Fmin,b}$ (2)

d_Fmax,b_ and d_Fmin,b_ are the median of the max. d_F_ and the median of the min. d_F_ of a budding cell.

This diameter is used to define the spherical segment, in which the bud can be hidden.

The curvature of a circle is determined according to (Eq. 3):

$y=\sqrt{2rx-x^{2}}$ (3)

in which r is the radius of the circle (r_m_) and x a certain distance in between 0 and r. In this case, x will be the radius of the daughter cell (r_d_), and y the distance from this point to the curvature (y_d_).

If y_d_ is known, the height of the spherical segment (h_ss_) and its radius (r_ss_) can be calculated (Eq. 4 and 5).

$h_{\mathrm{ss}}=d_{max,nb}/2-y_{d}$ (4)

$r_{\mathrm{ss}}=d_{max,nb}/2-r_{d}$ (5)

The surface jacket is determined with (Eq. 6):

$M=\pi\left( a^{2}+h^{2} \right)$ (6)

Substituting the formula with the relationships shown above, the area, in which a daughter cell is hidden by the mother cell (A_hd_), can be determined according to (Eq. 7):

$A_{\mathrm{hd}}=\pi\left( r_{\mathrm{ss}}^{2}+h_{\mathrm{ss}}^{2} \right)$ (7)

Finally, the relation of the total surface of the mother cell (S_m_) and the A_hd_  will provide the probability of false recognition of budding cells (X_Fnb_ ) as non-budding cells caused by this artifact (Eq. 8).

$X_{\mathrm{Fnb}}=\frac{A_{\mathrm{hd}}}{S_{m}} X_{b}$ (8)

Figure S2


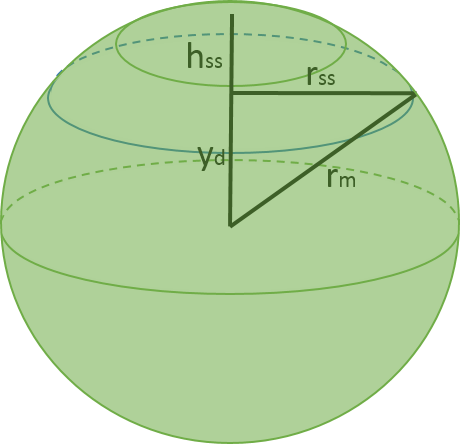


Scheme of a spherical cap used to evaluate the hidden area, where a bud can be located.

Figure S3


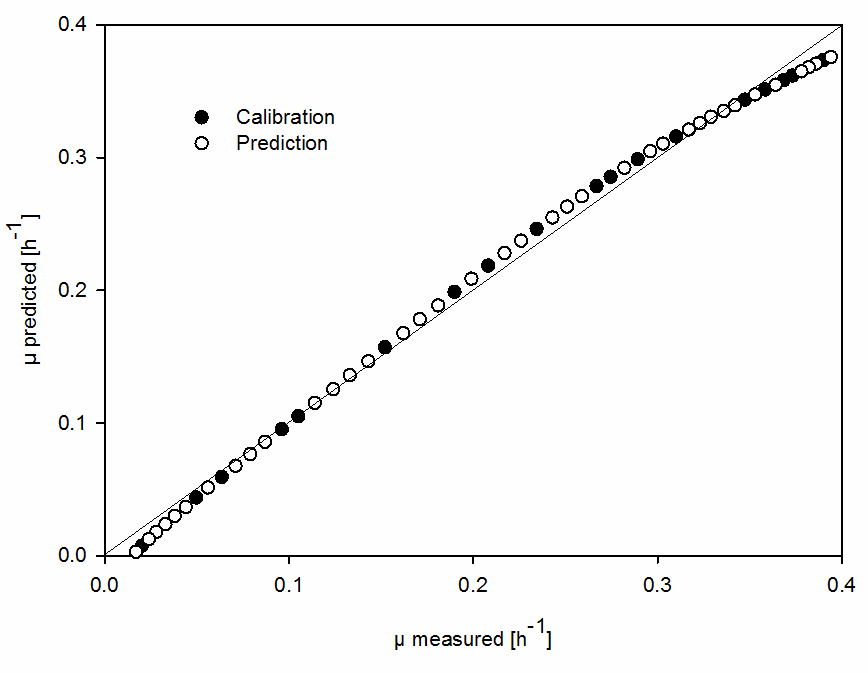


Linear correlation between the growth rate (µ), measured *off line* (using OD measurements), and predicted using the *on line* BI obtained through ISM and cross-calibration. Values used for calibration and prediction are depicted.

Figure S4


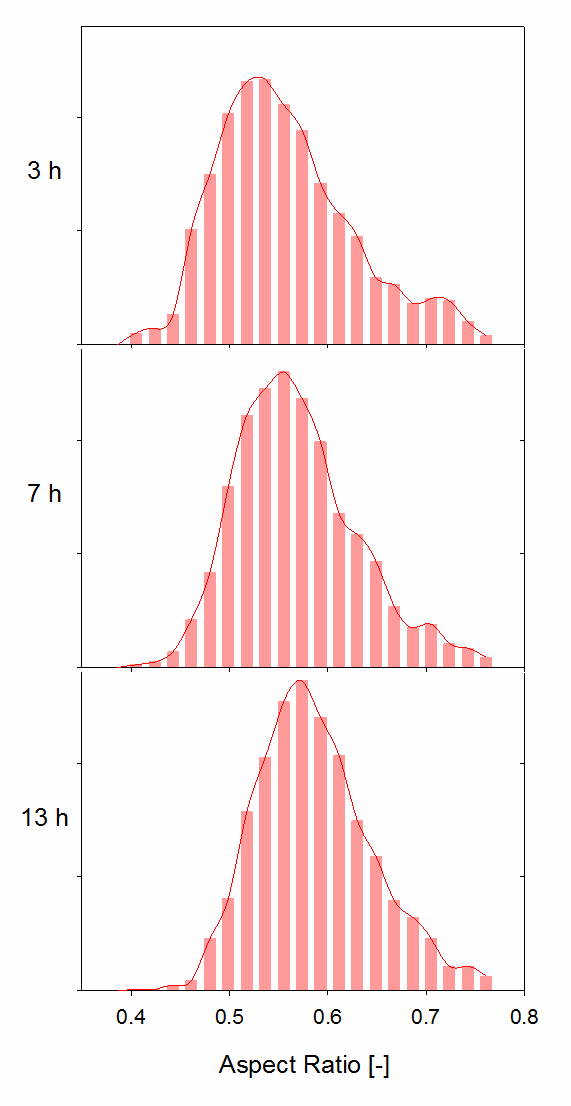


Single-cell frequency distribution of the AR of the budding cells during the cultivation time.
